# ‘Its many workers and subscribers feel that their services can still be of benefit’: Hospital Leagues of Friends in the English West Midlands, c. 1948–1998

**DOI:** 10.1093/shm/hkad031

**Published:** 2023-06-06

**Authors:** Gareth Millward

**Affiliations:** Danish Institute for Advanced Study, SDU, Odense, Denmark; ISKHK, Syddansk Universitet, Campusvej 55, 5230 Odense M, Danmark

**Keywords:** voluntarism, health, NHS, fundraising, hospital

## Abstract

Leagues of Friends are charities that provide ‘personal service to patients’ and ‘supply hospitals with equipment not likely to come from the budgeting of authorities’. Hundreds continue to exist, and many trace their origins to before the NHS’s foundation in 1948. Despite the rich and growing historiographies of voluntarism and the NHS, Leagues have received little attention. This article uses case studies of Leagues in the English West Midlands to show how ‘friendship’ symbolised the relationship between local NHS institutions and the communities they served. The cases show that voluntarism in British healthcare has not always been based around activism and consumerism, two areas that recent scholarship has rightly highlighted, especially from the 1960s. This allows historians to interrogate the regional and local differences within, ostensibly, a highly centralised national health system.

In a 1949 promotional leaflet, The Friends of the Children’s Hospital Birmingham urged local citizens to support their new organisation. The Brick League, which had fundraised and supported the patients and staff at the Hospital during the interwar period, disbanded with the introduction of the National Health Service (NHS) on 5 July the previous year. With the state taking control of staffing, treatment and capital projects at the Hospital, the Brick League no longer saw a future for itself—at least not in its pre-NHS form. The leaflet told readers that the new League of Friends’ activities would ‘vary from time to time according to the requirements of the Hospital, but it will aim at giving voluntary service and supplying those extra comforts for patients which are not provided by the Ministry of Health’. Even though the Brick League might be defunct, ‘its many workers and subscribers feel that their services can still be of benefit’.^1^

This continuity with 1930s voluntary service was not unique. Hundreds of Leagues of Friends were founded across Britain in the early years of the NHS and joined the National Association of Leagues of Hospital Friends (NALHF). Their efforts were often coordinated by members of groups that had been attached to pre-1948 hospitals, but whose remit and activities had been superseded somewhat by nationalisation. This article uses records and publications of Leagues of Hospital Friends that have been deposited in county records offices and libraries in the English West Midlands region^2^ to show how these organisations formed a link between NHS institutions and the local communities they served. These organisations are detailed in Table 1. This approach provides a sample of 15 Leagues from a range of institutional types and geographical locations—from the rural long-stay psychiatric hospital in Weston under Wetherby in rural Warwickshire, through the ex-workhouse general hospital in suburban Solihull, to the prestigious Royal Orthopaedic Hospital in South Birmingham. The article argues that these hitherto overlooked sources give insight into how voluntarism endured—in the form of ‘friendship’—even after the state took responsibility for local institutions’ finances.^3^ By focusing on League of Friends’ voluntary and fundraising activities, we see the gendered labour involved in their efforts. Importantly, we also see how Leagues acted as conduits between the NHS and their local communities through ‘friendship’: directly through membership and providing entertainment; and indirectly through their strong ties to other non-governmental organisations (NGOs) in their regions.

**Table 1.** West Midlands’ Leagues of Friends material used in this article

	County	Years active	Main hospital	Main hospital type	Archival holdings
Friends of Pershore Cottage Hospital (later: of Pershore Hospital)	Worcestershire	1935–present	Pershore Cottage Hospital	Cottage Hospital	WAAS (1935–2012)
Friends of Solihull Hospital	Warwickshire, Solihull	1953–present	Solihull Hospital	Ex-Poor Law general hospital	n/a
Penn Hospital League of Friends	Staffordshire, Wolverhampton	1980–97^a^	Penn Hospital	Cottage Hospital, geriatric specialism	Ephemera, WCA (1997)
Royal Hospital League of Friends (later: The League of Friends of the Royal Hospital, Wolverhampton)	Staffordshire, Wolverhampton	1955–96	Royal Hospital (closed 1996)	City general hospital	WCA (1962–95)
Stallington Hall Patients’ and Friends’ Association (later: Stallington Hall League of Friends)	Staffordshire	1953–97^a^	Stallington Hall (closed 1997)	Children’s learning disability hospital	STAFF (1964–84)
The Friends of the Warneford Hospital and Royal Midlands Counties Home (Royal Leamington Spa)	Warwickshire	1957–93(?)	Warneford Hospital (closed 1993)	General hospital	WCRO (1969–89)
The League of Friends of Birmingham Children’s Hospital (later: of Diana Princess of Wales Children’s Hospital, Birmingham)	Warwickshire, Birmingham	1948–2015^c^	Birmingham Children’s Hospital	Prestigious specialist hospital	BAH (1948–78)
The League of Friends of Central Hospital and Leigh House^b^	Warwickshire	1950(?)–95(?)	Central Hospital, Hatton (closed 1995)	Large mental health hospital	WCRO (1961–86); CARC (1961–71)
The League of Friends of the General Hospital, Birmingham	Warwickshire, Birmingham	1957–95(?)	Birmingham General Hospital (closed 1995)	City general hospital	BAH (1953–88)
The League of Friends of the Robert Jones and Agnes Hunt Orthopaedic Hospital (Oswestry)^c^	Shropshire	1964–present	Robert Jones and Agnes Hunt Orthopaedic Hospital	Prestigious specialist hospital	SA (1963–2006)
The League of Friends of the Royal Orthopaedic Hospital, Birmingham	Warwickshire, Birmingham	1975–2009^a^	Royal Orthopaedic Hospital	Prestigious specialist hospital	Ephemera, BAH (1980s)
The League of Friends of the Rugby Hospitals (later: The Friends of St Cross)	Warwickshire	1954–present	Rugby St Cross Hospital	Medium-sized general hospital	WCRO (1953–86)
The League of Friends of the Shipston on Stour Hospitals (later: of Shipston on Stour Ellen Badger Hospital)	Warwickshire	1973–present	Ellen Badger Hospital	Cottage hospital	WCRO (1973–96)
The League of Friends of Warwick Hospital	Warwickshire	1954–present	Warwick Hospital	General hospital	WCRO (1954–84)
Weston Hospital League of Friends	Warwickshire	1972–95	Weston Hospital (closed 1995)	Learning disability hospital/‘colony’	WCRO (1975–95)

*Notes*: BAH: Birmingham Archives and Heritage; CARC: Coventry Archives and Research Centre; SA: Shropshire Archives; STAFF: Staffordshire Records Office; WAAS: Worcestershire Archive and Archaeology Service; WCA: Wolverhampton City Archive; WCRO: Warwickshire County Record Office.

^a^Date taken from last filed accounts with the Charity Commission.

^b^Contained a semi-autonomous sub-branch based in Kenilworth, Warwickshire.

^c^Contained several sub-branches, including ones in Shropshire and Herefordshire.

*Source*: compiled from various archive holdings, catalogues, local histories and Charity Commission data.

The article further argues that the evidence from the West Midlands demonstrates that friendship manifested in different ways depending on the institution that Leagues of Friends represented. Pre-war voluntary networks, existing facilities on 5 July 1948, specialisms and prestige all had a significant effect on the foundation date, activities and, in some cases, decline of their Friends.

The material gathered and outlined in Table 1 contains numerous procedural documents and evidence of the Leagues’ activities, but it comes with some caveats. Each group had different approaches to both recording and preserving their materials, meaning some have very detailed correspondence, sub-committee minutes and publications covering many decades, while others only have a select number of documents relating to a narrower time frame. In particular, the Leagues of the Royal Orthopaedic and Solihull had little material (in the case of the latter, no material) available in public accessible archives, with much of the analysis refracted through secondary histories written by local historians. The time frame for which this material is accessible and its location are detailed in the rightmost column of the table. Furthermore, it is clear that there were many more hospitals with associated Leagues in the West Midlands in the second half of the twentieth century than are detailed here. Nevertheless, there is enough material here to provide a useful overview of different types of hospital and how their activities changed over time. It also provides an example of an approach that could be applied to other areas of the country.

Despite their proliferation after 1948, historians have shown remarkably little interest in Leagues of Friends. As George Gosling’s work on pre-war philanthropy, Jennifer Crane’s on NHS activism and the numerous references to volunteering in the oral history interviews conducted by the *NHS at 70* project show, there is continued historiographical interest in this long-standing—albeit constantly changing—relationship between health authorities and forms of collective and individual voluntary action.^4^ Yet Friends rarely feature. It is over 60 years since John Dodd published a booklet on voluntary effort in British and American hospitals through the British Hospitals Contributory Scheme Association and nearly 50 since Dame Leslie Whateley released her history of NALHM. Both authors had direct interests in the continuation of contemporary voluntary efforts.^5^ In January 2019, the only peer-reviewed academic research Deborah Davidson and her co-authors could find was a 1960 essay by E. W. Cooney commissioned by the Institute of Community Studies.^6^ But this lack of scholarly interest cannot be explained by asserting Leagues were unimportant, uninteresting or simply relics of forms of obsolete pre-war voluntarism. 175 Leagues were invited to NAHLF’s first meeting.^7^ In 2013, membership had risen to an estimated 1,500, and by 2021 NALHF (now rebranded as Attend) claimed to represent 29,000 volunteers across the UK.^8^

Importantly, the type of activity that Friends engaged in is significant. Histories of post-war voluntarism in British hospitals and healthcare have tended to focus on activism and consumerism. Investigations into these types of organisations have revealed much about the relationship between British citizens and their health services. Crane has shown how campaigns to save hospitals reflected the strength of feeling from local communities towards the institutions based in their towns, while Alex Mold’s research on patient–consumers has demonstrated how citizens organised to press for their rights and reshape health care services better towards their needs.^9^ These histories, however, tend to begin in the 1960s with the rise of new types of NGOs and a consumer rights culture, with an assumption that there was little engagement between the state and voluntary action in the NHS during the 1940s and 1950s. This is a fair conclusion for those interested in concepts and language such as ‘patients’ rights’, consultative bodies such as Community Health Councils and the growth of national organisations that directly lobbied governments.^10^ But it obscures day-to-day activity at the local level around less inherently ‘progressive’ causes. Indeed, with perhaps the exception of Robert Piggott’s work on the Church and the NHS,^11^ when historians have engaged with the history of small-c conservative groups in the NHS, these tend to focus on professional networks,^12^ or reactionary campaigns against specific policy areas such as sex education and family planning.^13^

Leagues of Friends are important because they were a continuation of forms of voluntarism that had their roots in the town associations and voluntary hospitals of the interwar period.^14^ Crucially, they did not disappear as the activist and consumerist groups became more visible. This local activity is significant. As John Glasby notes, British historiography’s focus on the *national* story of the NHS reflects, in part, its unusually high level of centralisation compared to other states; but this approach fails to account for—or even recognise—the local relationships that administrators at the regional and institutional level had with patients and well-wishers. Moreover, the NHS was not monolithic. There was significant variation between institutions and regions.^15^ These organisations are therefore an excellent window into how different parts of the UK expressed their relationship to the NHS as simultaneously a national and a very local institution embedded, via the hospital, in the community.

The neglect of Leagues also presupposes that activism and consumerism were more important (or, at the very least, more interesting) forms of voluntary activity than the—as Beaumont describes Women’s Institutes, Mothers’ Union and Townswomen’s Guilds—‘conservative, middle-class and mainstream’ organisations that had their roots earlier in the twentieth century and which did not disappear with the advent of the Beveridgean welfare state.^16^ Similarly, as Laura Balderstone’s work on middle-class suburbia has shown, pre-war voluntary practices—and the sense of community that came from them—have continued throughout the twentieth and into the twenty-first century.^17^ The continuation and evolution of this sort of voluntarism after the War can be traced through Leagues of Friends, even in an area where, ostensibly, the state had taken over from private and mutual forms of service provision.^18^

These forms of voluntary activity were expressed through ‘friendship’. Though no definitive definition of this concept was ever given, it was clear through the NALHF and the archives of individual Leagues that those involved in these organisations knew what it meant. Often it took the form of visiting, volunteering, gift-giving and fundraising on small- and large-scale projects. Sometimes friendship could result in political activism, such as when a local hospital was under threat—though it was much more likely to show itself through coordinated activity aimed at improving the quality of life of patients and staff. To explain how Leagues engendered friendship and to make the case for further research on these organisations, this article begins by explaining what twentieth-century Leagues of Friends were, what they did, where they came from, who was involved with them and how their development was affected by the longer history of institutional distribution. This provides the context to the second section which explores how ‘friendship’ manifested through voluntary labour and fundraising. Here we see the gendered nature of the work being performed in the name of friendship, as well as the different tactics and capacities of Leagues representing hospitals of different types and locations. The article ends by demonstrating that, while the archives of many Leagues exist because of their eventual dissolution, a good number did survive. The differences can again be understood through ‘friendship’, allowing some groups to adapt to adverse circumstances, especially when major fundraising activities became the demesne of other bodies after the 1980s.

## Leagues of Hospital Friends

Leagues of Friends are, according to Cooney, organisations which exist to provide ‘personal service to patients’ and ‘to supply hospitals with equipment not likely to come from the budgeting of the authorities’.^19^ This approach was reflected in the Warwick group’s constitution, which declared itself established:

(1)To provide a link between Warwick Hospital and the community which it serves;

(2)To promote and maintain by means of voluntary service, the interest of the public in the work [of the Hospital]

(3)To raise funds for and to provide amenities, facilities, comforts, entertainments, etc. [...]

(4)To raise funds [...} for the provision of items of capital or other expenditure which, in the opinion of the Committee, are urgently required and which might not normally be deemed to be the responsibility of the State but which the Hospital Authorities will be unable to provide for an indefinite period of time owing to the lack of official funds or because other items [...} have been granted a higher degree of priority.^20^

The wording here was almost verbatim in Rugby’s constitution, and the repetition of certain phrases across other groups in Charity Commission data suggests that NALHF had a proforma constitution for its members to amend according to their specific needs.^21^ Indeed, in 1948 the British Hospitals Association provided a very similar ‘Draft Constitution’ in the hope of encouraging the formation of such Leagues.^22^

The sheer size of the NHS budget meant that Leagues were not able to raise the kind of money that would make a material difference to day-to-day service provision. Cooney estimated the total income of all Leagues was equivalent to around £1 for every £600 of Treasury expenditure on the NHS in 1960.^23^ In the financial year 2010/11, Attend claimed its members had raised £44.4 million—which would equate to approximately £1 for every £3,700.^24^ The discrepancy can be explained by the expansion of the NHS’s budget since the 1960s, but is also affected by the 1980 Health Services Act which allowed hospitals to fundraise for extra capital and equipment expenditure. Whereas in the earlier period large campaigns might go through Friends’ accounts, after 1980 these monies could go instead directly to the hospital itself.^25^ Historian Frank Prochaska has been highly critical of the Act, arguing that it effectively made voluntary action an agent of the state.^26^ Still, as will be discussed, this was not necessarily fatal for League of Friends. The groups had close relationships with local state authorities well before 1980; and their ‘friendship’ activities continued well after.

Besides, a focus on gross income misses the point. It was never the intention that Leagues would augment hospitals’ running costs or provide treatments. The sums raised were certainly helpful to hospitals, and allowed them to improve the quality of decorations, patient and staff comfort and, in some cases, gain access to pieces of equipment that might otherwise be too costly for the NHS to prioritise or pay for the entire capital cost. These ‘frills’^27^ or ‘the trimmings’^28^ were, however, a means to an end. The Leagues’ core function was as *Friends*, not just of the hospital, but of the patients as well. Visiting and volunteering were key elements of the groups’ endeavours, often coordinated with other local organisations. Fundraising, too, involved voluntary labour coordinating the efforts of the hospital, the local community and various other NGOs. These activities would differ in form and scope according to the type of hospital and the capacity of the League in question.

This reflected Leagues of Friends’ origins from the networks formed around the pre-war voluntary hospitals. These institutions had been founded and were run on charitable donations, providing pay beds for those who could afford them and charitable care to those who could show they were in ‘genuine’ need (however determined by management). By the interwar period, the increasing costs of healthcare had affected the financial stability of many ‘voluntaries’. One solution had been the proliferation of contributory schemes that would guarantee access to affordable hospital care for working and lower-middle-class citizens. For small weekly payments, members would gain access to the hospital without the need to prove their moral rectitude and genuine financial distress, as well as having representation on management committees.^29^ Additional cash came from fundraising events and the solicitation of large legacies or grants from wealthy donors. The result of this activity was that there were well-established networks for both fundraising and volunteering around these hospitals.

The NHS obviated the need for the contributory schemes, as hospital care was now available free at the point of use to all. However, it did not entirely remove demand for mutual forms of insurance; nor did it destroy the presumed need for voluntary efforts and supplemental fundraising.^30^ The British Hospitals Contributory Schemes Association, which had represented the schemes attached to voluntary hospitals, immediately re-established itself with ‘(1948)’ added to its title.^31^ Meanwhile, the volunteers came to be represented by new charities—Leagues of Friends—many of which were direct descendants of the various ‘Ladies’ Guilds, Samaritan Funds, and the like’ that had previously coordinated fundraising and volunteering.^32^ NALHF began in 1949, with its first Chairman, Percy Wetenhall, being the Secretary of the voluntaries’ representative organisation, the British Hospitals Association.^33^ As discussed, The Friends of The Children’s Hospital Birmingham were immediately constituted out of the hospital’s pre-NHS ‘Brick League’.^34^ Likewise, individual stories of continuity emerge from the Midlands’ archives. Stella Edyvean-Walker was a committee member of Rugby’s Friends and had been a prominent campaigner in the pre-1948 Linen Guild and Ladies’ Committee attached to Hope Cross Hospital.^35^ She was also a Vice President of the League attached to Central Hospital in Hatton.^36^ Meanwhile, the Friends of the Cottage Hospital in Pershore had existed in a previous guise from the 1930s. When the League reformed in 1952, its records and many of its old members remained, including Evelyn Wilson who, when she retired as Treasurer in 1982, had been on the committee for 47 years.^37^

The leadership of these committees was, however, disproportionately staffed with, to quote Beaumont again, ‘conservative, middle-class and mainstream’ members. Hayes and Doyle have demonstrated how the contributory schemes were vehicles for middle-class sociability.^38^ Yet, as they also note, working-class voices were not absent. The growth of contributory schemes can only be explained by the vast increase in working and lower-middle-class citizens (alongside existing middle-class contributors) being willing and able to join. Yes, as Gorsky, Mohan and Willis show, most of these members simply wanted health coverage and were not interested in actively participating in the running of these organisations.^39^ At the same time, the success of large communal fundraising activities such as ‘flag days’ and ‘whist drives’ shows that local communities were willing to support for the voluntary hospitals and the organisations that existed to help them.^40^ Thus, while historians need to be mindful of the demographics in charge of Leagues and the inherent power dynamics therein, the amount of money and support for their activities must be seen as part of the wider story of voluntary activity across different sections of the British public.

Indeed, it is this background that explains how and why ‘Friends’ became established. With the direct financial requirement to support hospitals’ staffing, treatment and core capital costs removed, the need to provide the ‘frills’ and ‘trimmings’ remained. In turn this explains the disproportionate gender balance in Leagues towards women and feminised labour. Victoria Bates’ work on the long history of attempts to ‘humanise’ healthcare environments demonstrates that League activities such as visiting, decorating and organising activities for patients were designed to improve the atmosphere and general well-being of patients outside the biomedical treatments provided by modern drugs, equipment and trained professionals.^41^ As with the class element, this is not to say men did not participate in League activities. Yet the prevalence of reference to ‘ladies’ in League minutes, the emphasis on ‘service’ to the hospital and the activities that are discussed later in this article show the gendered nature of much of this work.

While this helps to explain the origins of the Friends in the West Midlands and the nation overall, it is important to note that not all hospitals were voluntaries before 1948. Indeed, only around 70,000 hospital beds in England and Wales were from the voluntaries, compared to 370,000 in the public sector.^42^ Therefore, existing voluntary networks and the capacities of the hospitals themselves could differ significantly. Poor Law hospitals were chronically underfunded. There is debate as to the extent to which the transfer of responsibility from Poor Law guardians to local authorities in 1929 improved their provision somewhat. As Powell and Levine argue, expenditure on these hospitals increased significantly over the 1930s; but this was disproportionately focused in the larger, more financially stable local authorities and on general hospitals. Care in long-stay institutions and in the psychiatric hospitals remained generally much lower.^43^ Regardless, the origins of these hospitals, as detailed in Table 1, are important to the biographies of the Friends that represented them. Their capacities and activities were affected by the pre-war voluntary groups (or lack thereof), the type of care provided in these institutions and the quality of the hospitals’ infrastructure in 1948.

This disparity was well understood in the 1950s. Minister of Health Iain Macleod and Lord (William) Beveridge both underlined the need for new associations to serve the old Poor Law hospitals and the mental institutions, recognising that these hospital types still experienced significantly less voluntary activity.^44^ Macleod discussed a group established in 1948 in Aberystwyth which had originally been designed to cover all the area’s hospitals but which decided to prioritise the old Poor Law infirmary in the town precisely because it was in such dire need of basic decoration and amenities such as chairs.^45^ In Rugby, a medium-sized market town, the majority of the League’s members appeared to come from networks around pre-war voluntary Hope Cross. But the League also saw the need to represent the ex-Poor Law St Lukes, which became increasingly focused on long-term care for elderly patients.^46^ Solihull’s Friends, formed in 1953, also had to buy rudimentary equipment for an ex-workhouse hospital that was considered out of date for the rapidly growing suburban town’s needs even before the War. According to local historian Joy Woodall, it spent a great deal of time campaigning for the Ministry to build a replacement—one it eventually got in 1994.^47^

It should be clear, however, that Leagues of Friends were not the only forms of voluntary action around NHS hospitals. Cooney found in 1960 that there were more Leagues in the South East of England than in the North or the Midlands, speculating that the groups grew quickest where an established and influential middle class saw the need for voluntary service of the specific type provided by Friends.^48^ As Table 1 shows, many of the Leagues in the West Midlands in this study were established well after 1948, even though 6 of the 16 were formed in the mid-1950s (a period which followed the government’s relaxation of rules about the separation of hospital management and voluntary activities). Nevertheless, all Leagues, regardless of their foundation date, had close ties to organisations such as the Women’s Royal Voluntary Service (WRVS), the Women’s Institute, Red Cross Cadets, Rotary Clubs, local businesses (management and workers) and many more besides. The sources considered in this article offer historians a view into how this voluntary activity was coordinated—but they should not be taken as the only forms of charitable work. Similarly, League-like activity was present around institutions even when a formal League had not been established. Shipston, for example, had been an informal network of volunteers that only officially organised in 1973 when the cottage hospital was in danger of closure.^49^ Meanwhile, the prestigious voluntary and teaching Royal Orthopaedic Hospital had found so much success with ad hoc local and national charitable efforts that it did not establish its League until 1975.^50^

Finally, NHS politics at the local and national levels affected the fortunes of Leagues after they had been formed. The psychiatric hospitals were greatly affected by policies of deinstitutionalisation. From the 1957 Royal Commission and Enoch Powell’s 1961 ‘water tower speech’ onwards, successive governments committed to reduce the NHS’s and social services’ reliance upon long-stay institutional care, moving people with mental illness and learning disabilities into the community.^51^ This accelerated in the 1980s and, as will be discussed, caused significant challenges, particularly for the Kenilworth sub-branch of Central Hospital Friends and Weston. Similarly, while Shipston’s Friends always had to fight against its hospital’s closure, Pershore was only pushed towards this kind of campaigning activity from the early 1980s when it became clear the Area Health Authority for Hereford and Worcester wanted to rationalise the smaller hospitals in Pershore, Evesham and Malvern.^52^ It is one of the ironies of using county records offices for evidence of League activity that these groups existed for many decades because they were attached to stable state institutions; yet the reason much of this material has been deposited is because those same hospitals were eventually closed due to strategic decisions by regional and national health authorities. Still, the range of types of hospital in Table 1—some of which, along with their Friends, are still active—gives us a broad base from which to discuss the various types of activity and how it changed over time.

## The Friends’ Activities

All the Leagues, regardless of size or the function of the hospitals they represented, placed great importance on their physical presence in their hospitals and on how volunteering materially improved the experience of being in hospital (and therefore the quality of treatment). Visiting—and the facilitating of visiting by others—was a core function of all the Leagues, albeit one with a clear gendered dimension.

The link between voluntary action and any fundraising was considered important to maintaining the quality of care in the hospital. ‘Friendship’, according to Weston’s Friends, was ‘one thing that did not show up on the balance sheet’,^53^ and though money was significant ‘service and personal contact was equally important’.^54^ This was integral to the foundation of many groups. Stella Edyvean-Walker had been part of the pre-NHS Lady’s Committee at Rugby Hope Cross, whose core function until it disbanded in 1951 was to provide visits and company to the patients.^55^ Visiting was given as a core function of the Shipston group in their first newsletter, imploring those with a car to chat to and make friends with patients.^56^ Kenilworth branch noted its ‘main efforts... centre on service to the patients’, including visiting and friendship.^57^ At the specialist hospitals, patients required significant medical interventions and, therefore, lengthy (or repeated) stays.^58^ For the Birmingham Children’s Hospital, friendship was also deemed necessary for the institution’s trainee nurses, who themselves might be away from family for extended periods.^59^ For those with larger budgets, visits could be coordinated with other gifts such as decorative flowers or Christmas presents.^60^ Indeed, Christmas was an important time for visiting and gift-giving in general. The Kenilworth sub-branch of Central Hospital even joked in the 1960s that its Father Christmas should have an ex-officio seat on the committee.^61^

Yet visiting took on a specific meaning to Leagues affiliated with long-stay hospitals and convalescence homes. For those patients without families able to visit on a regular basis, the human contact that could come from volunteering was considered central to the Friends’ mission. Shipston highlighted the need for visitors at the home for elderly people in the village.^62^ Stallington, a hospital specialising in care for children with learning disabilities, declared that ‘the whole of the residents are represented by the... friends who visit them. The residents are our children, and it is up to us to try to make life as pleasant and as tolerable as we can’.^63^ Weston found that visiting could offer stability, especially at times where there was a large turnover of staff which took familiar faces away from residents.^64^

But such visiting came with obligations. Kenilworth sub-branch reluctantly had to scale back visiting in the mid-1970s owing to a lack of volunteers, the ageing hospital population and reliable information from Central Hospital about the patients’ hometown. It did not want to let residents down. Instead, it reconfigured its regular day trips to Kenilworth as events held at the Hospital, allowing those with restricted mobility to take part and allowing the ‘ladies’ to run them with less labour power.^65^ Similarly, Weston warned of the dangers of casually visiting a few times and then suddenly withdrawing. The committee argued that Weston’s patients’ disabilities meant that they could not understand why their new friends were suddenly no longer visiting, causing distress and feelings of rejection.^66^

While they were able to provide visits themselves, the Leagues were also concerned with facilitating visiting from loved-ones and family members. This could sometimes require direct volunteering. The general hospitals’ Leagues made much of their work alongside WRVS and other local groups of providing car journeys to patients and their visitors who had no other reliable means of getting to the hospital. This was less important for the long-stay institution where patient numbers were lower and other long-term arrangements could be made.^67^ True, car transport also became less important to general hospitals over time (even though it continued), being far less prominent in annual reports and AGM summaries of the year’s activities.^68^ This probably reflected higher car ownership and access to other forms of transport. Still, the ethos of enabling quick visiting was more important to the acute hospitals.

For those groups who could afford it, overnight waiting rooms were also considered invaluable to allowing loved-ones to visit patients more easily and for as long as they needed to.^69^ Warwick and Rugby were proud of the onsite facilities that they had furnished in the 1950s and continued to develop. Both noted that their hospitals’ accident and emergency departments often dealt with severe road traffic accidents on major through routes. A room in which visitors could wait, prepare basic food and possibly sleep was a great comfort to those who might live many miles away and need to quickly get to their loved-ones’ bedside. It was cheaper and more convenient in a crisis than a hotel room, and thus remained important even as vehicular access to the hospital improved.^70^ When the Royal Hospital Friends in Wolverhampton disbanded in 1996, they considered that their overnight ‘flat’, built in 1962, their biggest success. Upon closure, the League of Friends and hospital management at New Cross Hospital (which was to absorb and replace the Royal) agreed to build a new overnight room and dedicate it to the Royal.^71^ RJAHOH’s committee also spent significant sums adapting a building to suit this purpose.^72^ The long-stay institutions in this paper did not have such critical care needs.

As has already been hinted at through mentions to Edyvean-Walker and the WRVS, this visiting work was largely undertaken and coordinated by women. Shipston’s League had 16 local visiting organisers at the time of formation, of which 15 were women. The 16th held the post jointly with his wife.^73^ Even in 1994, there were two husband and wife teams and a single man with a post in his own right.^74^ These coordination roles often involved liaising with other voluntary organisations, which also partook in visiting. Here too, there is a recurring, gendered theme. Weston’s friends coordinated the volunteering rotas with the WRVS, while AGM minutes also thanked the Ladies Auxiliary of the Licensed Victuallers Association for their long-term support.^75^ As Eve Colpus has demonstrated in the context of the interwar period, the idea of ‘women’s service’, and the gendered and classed labour they performed in their volunteering efforts, constantly changed. The move towards volunteering based on ‘the mutuality of self-fulfilment and community development’ is certainly evident in the visiting work interwar Friends and Linen Guilds.^76^ After the Second World War, such community work is infused with contemporary attitudes towards care. This care remained feminised.^77^ It also must be seen in a political climate in which visitation was seen as psychologically beneficial, even a right for visitors and those being visited. Beaumont demonstrates, for example, the pressure the Women’s Institute put on the government in 1950s to give parents the right to visit their children in hospital.^78^ Visiting was thus seen as important—but also as a task for the League’s women.

The juxtaposition of women as volunteers of labour in the hospitals and men as donors of professional or practical skills is evident in the constitution of many of the League’s committees. When a body was formed to set up the Birmingham General Hospital’s Friends, separate tasks were made for contacting ‘ladies’ organisations’ and ‘mens’’.^79^ At the first AGM, the Chairman, Treasurer and Secretary were all men, although the 14 non-officer committee members were equally split.^80^ At Rugby in the late 1960s, the Chair and Treasurer were men, although over half the non-officer members of the committee were identified as ‘Mrs’ or ‘Miss’.^81^

This split is evident in the organisation of activities that could both raise funds and act as a conduit to local people. Fairs, fetes and flag days had long been used by voluntary hospitals and the tradition had continued into the NHS era. They simultaneously raised money and engendered attachment to the institution from those who attended and, crucially, those who volunteered to organise the events.^82^ Fairs continued to be important to Leagues’ financial and volunteering rhythms across the twentieth century. Weston put a lot of voluntary effort into their annual Christmas Fair and Flag Day, which provided the bulk of their annual income in the 1980s.^83^ Penn, established as late as 1980, used its Summer Fair as the rallying point for the year’s activities.^84^ Even the specialist hospitals, who had access to many larger funding streams, highlighted fairs in their publicity material as examples of voluntary action and the strength of public attachment to their institutions.^85^

Indeed, fetes were clearly so much a fact of hospital life that the producers of ATV’s *Emergency Ward 10* felt the need to include one in their programme. They approached Warwick’s Friends in 1964 to organise ‘the Oxbridge Hospital Fete’ as both a fundraiser and for ATV to record footage. The Friends considered this an opportunity to demonstrate ‘the importance of the part which leagues of hospital friends now play in the country’s hospital service’.^86^ That year’s fete raised £1,942, or 76 per cent of the League’s annual income (versus £811, or 60 per cent, the previous year).^87^ Knowing the drawing power of broadcast celebrities, Weston secured actor Norman Painting to open its 1978 Christmas Fair, better known to the public as Phil Archer from *The Archers*, a BBC Radio 4 serial recorded in the Midlands.^88^ This proved more successful than attempts in 1975 by a committee member who had hoped to use her contacts to book the chimpanzees from the PG Tips television advertisements. Alas, the chimps had grown too big to perform.^89^ The Royal Orthopaedic was also able to bring in even bigger celebrities than an Archer. *The Good Life* star Felicity Kendall—who had grown up in Solihull next door to the architect of a proposed training centre, and whose mother had been treated at the Hospital—made a 10-minute appeal on the BBC in February 1984. It raised £300,000.^90^

These fundraising projects involved the entire League as well as organisations, businesses and individuals from outside. Weston’s AGM minutes note the help of the Kenilworth and Leamington Carnivals, the Red Cross Cadets, the Kenilworth Drama Group and MENCAP.^91^ Central Hospital in Warwick thanked St John Ambulance, Rotary Clubs, Round Tables, apprentices’ organisations, unions and churches. They were clearly proud of these links, lending weight to their claim that the League had ‘now become part of the Hospital’.^92^ When the Chairman at Birmingham Central Hospital’s League retired in 1961, he noted these links to other local organisations, and hoped that it further its progression ‘to form a bridge between the highly technical service of the Hospital and the Public’.^93^

Some of the activities within this, however, were clearly gendered. For example, male butchers donated meat and expertise for ‘hog roasts’ in Warwick, supervised by male volunteers.^94^ Men also went onto the street with barrel organs to drum up donations for the hospitals.^95^ Given that men were often in the positions of Chairman and Treasurer, much of the financial work also came to them. An example of this is the Stallington Hydrotherapy Pool Appeal Fund, set up as a sub-group for their large campaign in the 1980s. In a meeting with stakeholders involving 13 people, the only women were the two ‘joint secretaires’ of the sub-group.^96^ This reflected and reinforced the gendered professional roles of those in attendance—hospital administrators, architects and accountants. The same dynamics were at play in Rugby, where one of the leading figures in setting up the league was Norman Edyvean-Walker (Stella’s husband), a local lawyer who had sat on, and later chaired, the local hospital board.^97^

Meanwhile, women—invariably referred to as ‘ladies’—were often found running smaller fundraising efforts, usually designed to allow community members or patients to participate. They were seen providing flowers, staffing shops, running ‘knit-ins’ and organising tea parties.^98^ Larger events sometimes required their organising efforts, but these were also around more feminised activities, most notably dances (or ‘Balls’) and entertainments for the nursing staff.^99^ The divide was so obvious among The Royal’s Friends in Wolverhampton that the AGM minutes often paid tribute to ‘the “Committee Husbands”’ for their help at events while the ‘wives’ ran the show.^100^ To borrow from Tronto and Fisher’s model of care, the masculine role was ‘care giving’ through raising money, sitting on committees and organising the Leagues’ bureaucracy. But it was women who ensured the reciprocal relationship of ‘care-receiving’ by embedding their voluntary activities in ways that involved local people and the patients themselves, simultaneously able to adapt to the needs of this constituency and building the emotional links of ‘friendship’ that the organisations’ mission statements demanded.^101^

Just as these fundraising elements engendered friendship, so could a sense of attachment to the League and the hospital raise more funds. The most obvious example of this was through membership fees, although it is worth stressing that hospitals had different capacities in this regard. In 1978, for example, Warwick made £84 from subscriptions versus £60 in Weston. This was dwarfed by the £775 amassed by Pershore and £1,800 at RJAHOH.^102^ These differences were also evident in the one-off donations and legacies that the groups attracted. Stella Edyvean-Walker used her social connections and artistic talents to auction her watercolours. In 1 year, she raised £1,200 for Rugby’s Friends which was put into a trust fund.^103^ Warwick’s 1971 accounts show donations from the Lockheed Employees Charity Fund, the Mayor and a local charity football competition.^104^ The Royal Orthopaedic, being built on land owned by the Cadbury estate, regularly received large donations and support for its big projects from the family.^105^ While smaller groups did have connections to local groups that resulted in donations, this was not a large or reliable source of income for them. The only exception was a tactic used by both Weston and Stallington of writing to the next of kin of new long-stay patients—a less viable (or necessary) approach for the shorter-stay hospitals. The recipient of the letter was told of the fundraising and volunteering work the League did to make Hospital life more pleasant and asked if they would be willing to donate either time or money to help the League’s endeavours. Weston noted some success in bringing in more donations in this way in 1987, though discussions about volunteering and committee member shortages elsewhere suggest it was not as successful at soliciting labour.^106^ Even here, they were at a disadvantage. The private wards at the Royal Orthopaedic were useful to the Hospital Chaplain Rev. Collyer, who used his position to encourage all patients (but especially those with means) to donate to the hospital that had cared for them.^107^

Having raised all this money, it had to be spent. Sometimes this was done as an indirect ‘advertisement’ for the work of the League, hoping to solicit more interest and money further down the line. At other times, money was put into projects that would directly generate opportunities for more voluntary and/or fundraising work. Warwick, for example, strategically focused on buying a series of large items year after year. Rugby and the Royal Orthopaedic collated lists from hospital workers and management of potential donations and bought several gifts each year for patients and staff (including medical equipment), which it then used to promote itself to those same groups.^108^ Warwick and Solihull used their incomes and track records to demonstrate to management and regional NHS boards that they could cover a significant proportion of capital investments such as sunrooms or entertainment halls. Authorities approved and part-finance these projects, even when the money had not yet been raised.^109^ This allowed them to advertise and run larger campaigns with a clear end goal in sight. Pershore, too, was able to convince local authorities to extend services at the cottage hospital, provided the Friends could produce the bulk of the capital expenditure.^110^ Those without the turnover of the larger organisations, such as Weston, deliberately focused on single projects in the low four-figure range, devoting its remaining energies to what it could provide at no or low cost. According to their Chairman, by purchasing a single, relatively large item each year, the Friends could point to a visible gift which would provide better opportunities to promote themselves to the local community—as opposed to an ‘invisible project’, such as providing a holiday fund for patients, which, though welcomed, was much more difficult to exploit in the media.^111^

Still, a significant amount of investment was made into facilities and equipment for patients and staff to extend volunteering opportunities. For example, buses were common gifts. They provided a material benefit for patients—the ability to get away from the hospital for short trips to resorts, theatres or local attractions—but also fitted Leagues’ volunteering ethos. Stallington decided to undertake two large, multi-year fundraising campaigns, including one for a specially adapted minibus (the other for a hydrotherapy pool).^112^ In this way, Friends could not only provide transport, but also organise and chaperone any trips made with the vehicle.^113^ Weston, too, had raised funds for a minibus soon after it was founded, while Leamington had invested in a fleet of three.^114^

Shops and other commercial operations also made an explicit link between providing facilities for the hospital, raising money for the League and requiring volunteer labour. A ‘tuck shop’ opened in Weston in 1981, staffed by WRVS and League volunteers. ‘The Chairman welcomed this idea’, it was minuted at the 1982 AGM, ‘as it would mean the League being more actively involved with the patients than just a fund-raising organisation’.^115^ A new ‘club room’ allowed the League (and other groups) to arrange visits and activities with the patients in a dedicated space.^116^ Meanwhile, Warwick built a canteen with help from the Regional Hospital Board in 1956, coordinating a rota with the local WRVS and Women’s Institute branches to keep it open, while Stallington’s tuck shop opened in the mid-1970s.^117^ Where a static shop was not appropriate, trolley services were popular, such as the one in Shipston.^118^ The Royal Hospital League of Friends in Wolverhampton was so successful with its trolley service that travelled from ward to ward selling refreshments that it registered itself as a business and provided significant funding to the league via its profits.^119^ RJAHOH’s hospital shop was transferred to the League in 1965, and grew into a wheelchair accessible supermarket. It had permanent staff—including a ‘manageress’—but was kept running by volunteer labour, especially from WRVS and the Women’s Institute.^120^ Indeed, just as with the knit-ins and annual dances, these volunteer-led operations were, again, opportunities for feminised labour, facing the patients and local community.

## The Decline of the Friends?

As discussed earlier, one of the reasons Leagues of Friends’ documents are available in county records offices is because the organisations and/or their parent institutions no longer exist. Friendship was not always enough, therefore, to keep the charities operational. However, as seen in Table 1 and the research of Ellis Payne and her co-authors, many do remain.^121^ Furthermore, in attempts to save hospitals and in the processes of winding up Leagues due to planned closures, League of Friends re-emphasised the importance of friendship. Thus, these archives show us how friendship and the relationship between local communities and the NHS operated both in ordinary and extraordinary circumstances.

The fight to keep institutions open was stronger in the specialist and the general hospitals than in the psychiatric institutions. In part, this reflected practical considerations about the direction of travel of NHS policy and the commitment to deinstitutionalisation. ‘Rationalisation’ projects stood more chance of being overturned. The cottage hospitals were at most risk, here. Just as the Women’s Institute had pressured government for patient visiting rights in the 1950s, so could League of Friends show their friendship through supporting their institutions’ existence.^122^ Shipston’s Friends were formally established in 1973 to oppose the potential closure of the hospital and are still active in 2023.^123^ Pershore’s Friends, which in various forms dated back to at least the 1930s, found it too had to oppose potential closures in the 1980s. It was able to draw on local anxieties and support from Conservative (upper and lower case) parish councillors—the market town had lost its railway station, magistrates court and had its Post Office downgraded over recent years.^124^ However, Penn was less successful, despite organising a protest and petition against the decision to close it in 1992.^125^

It was not just the cottage hospitals who had to defend their institutions, however. The Royal at Wolverhampton unsuccessfully opposed plans to gradually transfer services to New Cross which had been rebuilt in the 1970s. As the wards gradually emptied over the 1980s, it too found its membership getting older and fewer in number. It tried to reorientate its activities. In 1988, one member argued ‘it is time to give money raising efforts a rest’ because ‘the aim of the League when founded was to give service’.^126^ But when the decision on its total closure was made in 1995, it dissolved the refreshment trolley business and, at an Extraordinary General Meeting in April 1996 announced the League would disband completely from the end of June.^127^

It was quite a different story at the Royal Orthopaedic Hospital. In 1992, Conservative Secretary of State Virginia Bottomley had ordered it to close by April 1994, but the decision was overturned. The hospital had several advantages over Weston and the Royal at Wolverhampton. The Royal Orthopaedic had a national reputation, as evinced by the Felicity Kendall appeal in 1984. It sat on land owned by the Cadbury family rather than the State, and so the resale value was unlikely to raise funds the local health authorities. It was also in a marginal Conservative parliamentary constituency. Furthermore, whether treatments were provided at the Royal Orthopaedic or elsewhere, the scheduled operations and medical interventions would still have to be provided somewhere within Birmingham’s NHS institutions. There was no ‘surgery in the community’ policy. Thus, a long, visible local campaign got the hospital a reprieve.^128^

It is therefore clear that larger national trends as well as favourable local circumstances were needed as well as friendship to keep an institution alive. At the psychiatric hospitals, knowing such circumstances were not on the horizon, the Leagues focused heavily on how they could be of use in the little time they had left. At Weston, patient numbers had decreased over the 1980s and 1990s. In 1994, the hospital closed. Similarly, the Kenilworth sub-branch of Central Hospital noted from the early 1980s that policies around new patients and long-term care had led to an ageing hospital population, which affected the activities they could perform. It found it especially difficult to coordinate physical travel to and from Kenilworth, and there was increasing overlap with events being organised by the hospital staff themselves. No further minutes exist after 1986, suggesting it disbanded, though there is no indication that the main branch also closed at the same time.^129^

As the 1990s arrived, it was clear that Weston had no future and the Friends re-evaluated their role. More emphasis was placed on visiting, especially given the high staff turnover at the Hospital and the low direct cost to the organisation.^130^ But fundamentally, the group had to consider their existence. Would they continue as ‘friends’, visiting ex-patients who were resettled in the community and providing new friendship to local disabled people? The League for Middlefield Hospital near Solihull had done this, deciding ‘to continue to support all the former residents of the Hospital out in the community homes’ and ‘to broaden their scope to include those who would come after the patients of the Hospital based on geographical location’.^131^ However, Weston did not feel it had the resources or the energy to do this. The group’s committee had been getting older, and the number of active members had been shrinking for years. Much like Ellis Payne *et al.* found with cottage hospital Leagues in the 2010s, most volunteers were recruited through personal connections and remained in post for many years.^132^ This small pool of potential members combined with an ageing committee meant the group did not feel able to undertake such a dramatic change in their operation. Residents were being housed in multiple Leamington properties and in other towns, making the potential catchment area rather diffuse.^133^ It was felt such ‘friendship’ was important, but could be better served by other agencies. Thus, having rejected the Middlefield model, the Friends greatly scaled back fundraising activities in 1992, focusing solely on volunteering.^134^ The group folded at the end of 1994, donating their remaining funds to MENCAP and their archives to Warwickshire County Records Office.^135^ As Chairman Mrs Y. Hall declared, ‘we hope to go out on a high note!’^136^

## Conclusion

This article has shown how the archival records of Leagues of Friends provide a window onto hospital-related voluntary action over the second half of the twentieth century. This ‘friendship’ represented the relationship between local NHS institutions and the community. By integrating this material into the longer history of British healthcare, we see that voluntarism did not disappear on 5 July 1948. Instead, a range of existing networks reformed into new organisations that engaged in significant fundraising and voluntary action well before—and well after—the rise of activist and patient groups in the 1960s. The West Midlands provides a wealth of archival material that show these processes in action in different settings, explaining how these related to national health policy and political developments.

Despite the proliferation of these organisations and the many continuities between them, this article has also demonstrated that each League had different fortunes that can be explained in large part by the history of the institution they represented. Much like the distribution of voluntary hospitals had been uneven and reflected existing inequalities across Britain from the mid-nineteenth century onwards,^137^ these organisations were also more likely to appear in places with pre-existing voluntary networks. If they did emerge around other types of institution, they tended to appear more slowly and were more limited in their ability to act due to the state of those hospitals. While this is not a predictive tool of exactly how and when a group might emerge or behave, it gives good guidance to historians seeking to explain developments in other institutions elsewhere in the British Isles. Moreover, it allows for future comparisons within the UK. If Cooney was correct about the distribution of Leagues and hospitals in the 1960s, for example, it would be instructive to see to what extent the patterns found in the West Midlands are replicated in regions such as the South East or in the Celtic nations.

For these reasons, this article is also a call for more research. The form of voluntarism Leagues represented is an overlooked element of the relationship between the post-war state and the public. Increased searchability and accessibility of digitised local newspapers will help track the activities of Leagues whose formal records have not survived. Quantitative work on analysing the records of the Charity Commission could be adapted to focus on Leagues of Friends providing an overview of changes in the number of charities and reported income. The *NHS at 70* project has already shown the possibilities for oral history in the history of British healthcare, as have the countless other important interviews with people involved in NGOs. Volunteers, patients, staff and hospital administrators would be valuable witnesses. Importantly, many Leagues from the 1940s and 1950s—much like the hospitals they serve—continue to operate across the country. Approaches could be made to access materials and draw upon their members’ experiences. Traces of League and other voluntary activity are also evident in the records of individual hospitals and regional administrative bodies which, although sometimes difficult to follow in the long-term because of several national and regional NHS re-organisations, are often to be found in depositories across the UK.

Through doing so, we will find that, as historical witnesses, the Leagues’ ‘many workers and subscribers... can still be of benefit’.

## Footnotes

1 Birmingham Archives and Heritage (hereafter: BAH): HC/BCH/1/13/27, Promotional Leaflet, 1949.

2 This study defines ‘West Midlands’ as the region (rather than the metropolitan county) according to the traditional statistical definition—the counties of Herefordshire, Shropshire, Staffordshire, Warwickshire, Worcestershire and, after 1974, West Midlands. See: Office for National Statistics, ‘Eurostat’, Office for National Statistics, n.d., https://www.ons.gov.uk/methodology/geography/ukgeographies/eurostat, accessed 6 August 2021; Central Statistical Office, *Annual Abstract of Statistics, No. 102* (London: HMSO, 1965), 342.

3 On this relationship with regard to Christian voluntarism and the NHS, see: Robert Piggott, ‘Hospital Sunday and the New National Health Service: An End to the “Voluntary Spirit” in England?’, *Studies in Church History*, 2022, 58, 372–93.

4 George Campbell Gosling, *Payment and Philanthropy in British Healthcare, 1918–48* (Manchester: Manchester University Press, 2017); Jennifer Crane, ‘“Save Our NHS”: Activism, Information-Based Expertise and the “New Times” of the 1980s’, *Contemporary British History*, 2018, 33, 1–23. Oral history interviews from ‘NHS at 70’ are available through the British Library online catalogue. See NHS Voices of Covid-19, ‘Voices from Our Archive’, *NHS Voices of Covid-19*, n.d., https://www.nhs70.org.uk/momentsofcare, accessed 6 August 2021.

5 John Dodd, *Hospitals and Health Services in Britain and the United States of America* (Bristol: British Hospitals Contributory Schemes Association (1948), 1961); L. Whateley, *Yesterday, Today and Tomorrow: History of the National Association of League of Hospital Friends* (London: Law & Local Govt., 1974). Dodd had been a key NALHF member in 1949. Attend, ‘Those We Remember’, *Attend*, n.d., https://www.attend.org.uk/node/1579, accessed 10 August 2021. Dame Leslie had been commissioned by prominent NALHF members. David Wood, ‘Forged in the Fires of Belief? An Exploration of Faith and Community Engagement in the Member Groups of Attend’, *Attend*, 2013, https://www.attend.org.uk/sites/default/files/Forged%20in%20the%20fires%20of%20belief_2.pdf, accessed 8 November 2021.

6 E. W. Cooney, ‘The Leagues of Hospital Friends’, *Public Administration*, 1960, 38, 263–72; Deborah Davidson *et al.*, ‘Analysis of the Profile, Characteristics, Patient Experience and Community Value of Community Hospitals: A Multimethod Study’, *Health Services and Delivery Research* 7, no. 1 (January 2019): 4, doi:10.3310/hsdr07010. Since then, there has been another: Angela Ellis Paine *et al.*, ‘Communities as “Renewable Energy” for Healthcare Services? A Multimethods Study into the Form, Scale and Role of Voluntary Support for Community Hospitals in England’, *BMJ Open*, 2019, 9, e030243.

7 Warwickshire County Records Office (hereafter: WCRO): CR3206/97, The National League of Hospital Friends, Promotional material, probably 1951 or 1952; Attend, ‘Who We Are’, *Attend*, n.d., http://www.attend.org.uk/about-us/who-we-are, accessed 6 August 2021.

8 Sally Carter, ‘Know Who Your Friends Are’, *BMJ*, 2013, 346, f1022; Attend, ‘Who We Are’.

9 Crane, ‘“Save Our NHS”’; Alex Mold, *Making the Patient-Consumer: Patient Organisations and Health Consumerism in Britain* (Manchester: Manchester University Press, 2015). See also: Jon Glasby, Helen Dickinson and Judith Smith, ‘“Creating NHS Local”: The Relationship between English Local Government and the National Health Service’, *Social Policy & Administration*, 2010, 44, 244–64; Lorelei Jones, ‘What Does a Hospital Mean?’, *Journal of Health Services Research & Policy*, 2015, 20, 254–56.

10 On these groups and their development, see: Matthew Hilton *et al.*, *The Politics of Expertise: How NGOs Shaped Modern Britain* (Oxford: Oxford University Press, 2013).

11 Piggott, ‘Hospital Sunday’.

12 Andrew Seaton, ‘Against the “Sacred Cow”: NHS Opposition and the Fellowship for Freedom in Medicine, 1948–72’, *Twentieth Century British History*, 2015, 26, 424–49.

13 For example: Hannah J. Elizabeth, ‘“If It Hadn’t Been for the Doctor, I Think I Would Have Killed Myself”: Ensuring Adolescent Knowledge and Access to Healthcare in the Age of Gillick’, in Jennifer Crane and Jane Hand, eds, *Posters, Protests and Prescriptions: Cultural Histories of the National Health Service in Britain* (Manchester: Manchester University Press, 2022), 255–80.

14 Martin Gorsky, John Mohan and Tim Willis, *Mutualism and Health Care: Hospital Contributory Schemes in Twentieth-century Britain* (Manchester: Manchester University Press, 2006).

15 Glasby, Dickinson and Smith, ‘“Creating NHS Local”’.

16 Caitriona Beaumont, ‘Housewives, Workers and Citizens: Voluntary Women’s Organisations and the Campaign for Women’s Rights in England and Wales during the Post-War Period’, in Nick Crowson, Matthew Hilton and James McKay, eds, *NGOs in Contemporary Britain: Non-State Actors in Society and Politics Since 1945* (London: Palgrave Macmillan, 2009), 59–76.

17 Laura Balderstone, ‘Semi-detached Britain? Reviewing Suburban Engagement in Twentieth-century Society’, *Urban History*, 2014, 41, 141–60. See also: Chris Moores, ‘Thatcher’s Troops? Neighbourhood Watch Schemes and the Search for “Ordinary” Thatcherism in 1980s Britain’, *Contemporary British History*, 2017, 31, 230–55.

18 Piggott, ‘Hospital Sunday’.

19 Cooney, ‘The Leagues of Hospital Friends’, 263.

20 WCRO: CR3399/1, Constitution, objects and rules of procedure, 1954.

21 WCRO: CR3206/97, Constitution, objects and rules of procedure, 1955.

22 Oxfordshire Health Archives, HO-1, British Hospitals Association, Hospitals Yearbook 1948, 54–56. I thank Stephanie Haywood for directing me to this source.

23 Cooney, ‘The Leagues of Hospital Friends’.

24 Attend, ‘Annual Review 2011’, *Attend*, 2011, https://www.attend.org.uk/sites/default/files/2011.pdf, accessed 25 August 2021. UK NHS nominal spending 2010/11 was £166 billion. Office for National Statistics, ‘Healthcare Expenditure, UK Health Accounts: 2018’, Office for National Statistics, 28 April 2020, https://www.ons.gov.uk/peoplepopulationandcommunity/healthandsocialcare/healthcaresystem/bulletins/ukhealthaccounts/2018, accessed 25 August 2021.

25 Some of these campaigns are covered below.

26 Frank Prochaska, ‘The State of Charity’, *gov.uk*, 18 September 2014, https://www.gov.uk/government/news/the-state-of-charity, accessed 19 October 2021.

27 Cooney, ‘The Leagues of Hospital Friends’.

28 WCRO: CR3206/97, All About the League of Friends of the Rugby Hospitals, n.d., probably 1967 or 1968.

29 S. Cherry, ‘Accountability, Entitlement, and Control Issues and Voluntary Hospital Funding c. 1860–1939’, *Social History of Medicine* 1996, 9, 215–33; Lynsey T. Cullen, ‘The First Lady Almoner: The Appointment, Position, and Findings of Miss Mary Stewart at the Royal Free Hospital, 1895–99’, *Journal of the History of Medicine and Allied Sciences*, 2013, 68, 551–82.

30 Gorsky, Mohan and Willis, *Medicine and Mutual Aid*, esp. Chapters 9 and 10.

31 Lord Beveridge, ‘The Role of the Individual in Health Service’, *British Medical Journal*, 1954, 2, 1371–3.

32 ‘Friends of Hospitals’, *British Medical Journal*, 1948, 2, 44.

33 ‘British Hospitals Association: Annual Meeting’, *British Medical Journal*, 1948, 2, 88.

34 BAH: HC/BCH/1/13/27, Promotional leaflet, c. 1949.

35 See WCRO: CR3206/97; WCRO: CR2745/62; EWDL Trust, ‘Our History’, *EWDL Trust*, n.d., https://www.edyveanwalkerlindop.co.uk/our-history/, accessed 10 August 2021.

36 Coventry Archives and Research Centre (hereafter: CARC): PA 1991/4/18, The League of Friends of the Central Hospital and Leigh House to subscribers, late 1969.

37 Comparing the minutes of the last 1948 and first 1952 meetings, one sees P. G. Feek, W. F. Pugh, Mrs Mumford, Betty Smithin and Canon Bark. See: Worcestershire Archive and Archaeology Service (hereafter: WAAS): 705:1336 BA12116/1, Committee meeting 10 June 1948; WAAS: 705:1336 BA12116/2, Committee meeting, 25 July 1952; WAAS: 705:1336 BA12116/7, *Berrow’s Journal* cutting, 6 May 1982.

38 Nick Hayes and Barry M. Doyle, ‘Eggs, Rags and Whist Drives: Popular Munificence and the Development of Provincial Medical Voluntarism between the Wars’, *Historical Research*, 2013, 86, 712–40; Nick Hayes, ‘Counting Civil Society: Deconstructing Elite Participation in the Provincial English City, 1900–1950’, *Urban History*, 2013, 40, 287–314.

39 Gorsky, Mohan and Willis, *Medicine and Mutual Aid*.

40 Hayes and Doyle, ‘Eggs, Rags and Whist Drives’.

41 Victoria Bates, ‘“Humanizing” Healthcare Environments: Architecture, Art and Design in Modern Hospitals’, *Design for Health*, 2018, 2, 5–19.

42 Allen Daley, ‘British Hospitals as They Were Before 1948’, *British Medical Journal*, 1960, 2, 758–63.

43 M. Powell, ‘An Expanding Service: Municipal Acute Medicine in the 1930s’, *Twentieth Century British History*, 1997, 8, 334–57; A. Levene, ‘Between Less Eligibility and the NHS: The Changing Place of Poor Law Hospitals in England and Wales, 1929–39’, *Twentieth Century British History*, 2009, 20, 322–45; Martin Gorsky, ‘Creating the Poor Law Legacy: Institutional Care for Older People Before the Welfare State’, *Contemporary British History*, 2012, 26, 441–65.

44 Beveridge, ‘The Role of the Individual in Health Service’; WCRO: CR3206/97, NALHF, Iain Macleod’s address to the AGM, 14 January 1953.

45 WCRO: CR3206/97, NALHF, Iain Macleod’s address to the AGM, 14 January 1953.

46 See Rugby’s records in: WCRO: CR3206/97-100.

47 Joy Woodall, *Gin, Ale and Poultices... Lasers and Scanners: Solihull Workhouse and Hospital, 1742–1993* (Birmingham: Joy Woodall, 1994).

48 Cooney, ‘The Leagues of Hospital Friends’, 266.

49 WCRO: CR3894, Newsletter, October 1973.

50 Zoë Chamberlain, *The Friends: The History of the Royal Orthopaedic Hospital’s League of Friends* (Studley: Brewin Books, 2003), esp. 23, 45–46.

51 Eustace Percy, *Royal Commission on the Law Relating to Mental Illness and Mental Deficiency, Cmnd. 169* (London: HMSO, 957); Vicky Long, ‘“Heading up a Blind Alley”? Scottish Psychiatric Hospitals in the Era of Deinstitutionalization’, *History of Psychiatry*, 2017, 28, 115–28.

52 WAAS: 705:1336 BA12116/8, Minute book 1983–86.

53 WCRO: CR3158/3/1, AGM, 15 January 1976.

54 WCRO: CR3158/3/2, AGM, 24 April 1989.

55 See the minute books of the committee from 1913 to 1951: WCRO: CR2745/60–2, 64.

56 WCRO: CR3894, Newsletter, October 1973.

57 WCRO: CR3174/1, Secretary’s AGM Report, 1976.

58 BAH: HC/BCH/1/13/26, Promotional leaflet, c. early-mid 1950s; BAH: HC/BCH/1/13/27, Promotional leaflet, c. 1949; Chamberlain, *The Friends*, 32–44.

59 BAH: HC/BCH/1/13/26, Promotional leaflet, c. early-mid 1950s.

60 See several mentions in Weston (WCRO: CR3158/3/1); Central Hospital (CARC: PA 1991/4/18); Warwick (WCRO: CR3399/2). On decoration in hospitals, see: Bates, ‘“Humanizing” Healthcare Environments’.

61 WCRO: CR3174/1, AGM, 15 May 1968.

62 WCRO: CR3894, Newsletter, October 1973.

63 Staffordshire Records Office (hereafter STAFF): D5562/12/1, Statement of accounts to 31 March 1965. The same wording was used in subsequent years up to 1970.

64 WCRO: CR3158/3/1, AGM, 29 March 1979.

65 The sub-branch’s remit was to focus on residents from (or with significant ties to) Kenilworth. WCRO: CR3174/1, Secretary’s Annual Reports for 1972 and 1974.

66 WCRO: CR3158/3/1, AGM, 13 April 1978.

67 Warwick boasts of its transport service in: WCRO: CR3399/1, Secretary’s AGM Report, 1956; Shipston asks for a car transport coordinator in: WCRO: CR3894, Newsletter, June 1976.

68 Warwick, for example, noted ‘demands for the car service have fallen off somewhat’ as early as 1962. WCRO: CR3399/1, Secretary’s AGM Report, 1962.

69 The importance of waiting rooms and their architecture had become more prominent in the NHS after the 1940s, especially in General Practice. See: Martin D. Moore, ‘Waiting for the Doctor: Managing Time and Emotion in the British National Health Service, 1948–80’, *Twentieth Century British History*, 2022, 33, 203–29.

70 WCRO: CR3399/1, Secretary’s AGM Report, 1956; WCRO: CR3206/98, Cousins memorandum to various District admins, 11 July 1979.

71 Wolverhamption City Archives (hereafter: WCA): DX-1030/1, Extraordinary General Meeting, 16 April 1996.

72 Shropshire Archives (hereafter: SA): MI 5524/1/18, Annual Report, February 1981.

73 WCRO: CR3894, Committee list, n.d., but probably 1973.

74 WCRO: CR3894, Newsletter No. 34, February 1994.

75 WCRO: CR3158/3/1, Committee meeting, 5 June 1978; AGMs for 1983, 1984 and 1987; WCRO: CR3158/3/2, AGM for 1993.

76 Eve Colpus, ‘Women, Service and Self-acutalization in Inter-war Britain’, *Past and Present*, 2018, 238, 197–232, quotation p. 199.

77 Sarah Chaney, ‘Before compassion: Sympathy, Tact and the History of the Ideal Nurse’, *Medical Humanities*, 2021, 47, 475–84; Joan C. Tronto, *Caring Democracy: Markets, Equality and Justice* (New York: New York University Press, 2013), esp. Chapter 3.

78 Caitriona Beaumont, *Housewives and Citizens: Domesticity and the Women’s Movement in England, 1928–64* (Manchester: Manchester University Press, 2013), 206–8. See also Mold, *Making the Patient Consumer*, 25–29, on The National Association for the Welfare of Children in Hospital.

79 BAH: HC/GH/6/6/1, Proposed League of Friends, Interim sub-committee, 24 June 1957.

80 BAH, HC/GH/6/6/1, First AGM minutes, 24 March 1958.

81 See WCRCO: CR3399/1 in general, especially minutes of meetings in 1968 and 1969.

82 Hayes and Doyle, ‘Eggs, Rags and Whist Drives’; Gorsky, Mohan and Willis, *Medicine and Mutual Aid*.

83 In 1990, the Flag Day and Christmas Fair accounted for 48 per cent of income, versus 37 per cent in 1986 and 43 per cent in 1980. See accounts in: WCRO: CR3158/8.

84 WCA: LS/LP3621, History of the Penn Hospital League of Friends.

85 BAH: HC/BCH/1/13/26, Promotional leaflet, n.d., early-mid 1950s; SA: MI 5524/1/1-34, annual reports 1964–96; Chamberlain, *The Friends*, 32–44.

86 WCRO: CR3399/1, Secretary to members, 5 March 1964.

87 WCRO: CR3399/1, Income and expenditure accounts for years ending 1963 and 1964.

88 WCRO: CR3158/3/1, AGM, 29 March 1979.

89 WCRO: CR3158/3/1, Committee meetings, 16 January and 17 March 1975.

90 Chamberlain, *The Friends*.

91 WCRO: CR3158/3/1, Committee meeting, 5 June 1978; AGMs for 1983, 1984 and 1987; WCRO: CR3158/3/2, AGM for 1993.

92 CARC: PA 1911/4/18, Chairman’s Report, AGM, 19 April 1972.

93 BAH: HC/GH/6/6/1, Fourth AGM, 2 May 1961.

94 First mention for Warwick is: WCRO: CR3399/1, Secretary’s AGM Report, 1958.

95 Kenilworth: WCRO: CR3174/1, Secretary’s AGM Report, 1968.

96 STAFF: D5562/1/64, Minutes of meeting, 6 May 1980.

97 WCRO: CR3206/97, No. 20 Group Hospital Management Committee meeting minutes, n.d., probably late 1952; EWDL Trust, ‘Our History’.

98 Kenilworth: WCRO: CR3174/1, Secretary’s AGM Report, 1963; WCRO: CR3206/97, All About the League of Friends of the Rugby Hospitals, n.d., probably 1967 or 1968; Shipston: WCRO: CR3894, Newsletter, June 1976; BAH: HC/BCH/1/13/26, Promotional leaflet, c. early-mid 1950s.

99 On this and the previous footnote, see: Birmingham General: BAH: HC/GH/6/6/1, Third AGM, 28 April 1960; Shipston: WCRO: CR3894, Newsletter No. 29, February 1989.

100 WCA: DX-1030/1, various AGM minutes, but especially 1979.

101 Tronto, *Caring Democracy*, 22–23.

102 WAAS: 705:1336 BA14159/2/6, Cash account for the year to 31st December 1978; SA: MI 5524/1/16, Annual Report 1979.

103 WCRO: CR3206/97; EWDL Trust, ‘Our History’.

104 WCRO: CR3399/2, Income and expenditure account year ended 31st December 1971.

105 Chamberlain, *The Friends*, esp. 52.

106 WCRO: CR3158/3/1, AGM, 29 April 1987. For Stallington’s experience, see: STAFF: D5562/12/1, Proforma letter, March 1974.

107 Collyer recounts this tactic—‘before data protection’—in Chamberlain, *The Friends*, 49–50.

108 See correspondence with Hope Cross: WCRO: CR3206/98–100; Chamberlain, *The Friends*, 32–44.

109 Similar projects occurred throughout the period, but see esp., WCRO: CR3399/1, W. A. James, Group Secretary, South Warwickshire Hospital Group (No. 14) to W. F. C. Leslie, Secretary, League of Friends of Warwick Hospital, 23 February 1959; Woodall, *Gin, Ale and Poultices*, 47.

110 See, for example, the refurbishment and extension of physiotherapy facilities across the 1970s and 1980s: WAAS: 705:1336 BA12116/7, *Berrow’s Journal*, 11 February 1983; WAAS: 705:1336 BA12116/5, *Evesham Journal*, 25 November 1976.

111 Direct quotation WCRO: CR3158/3/1, AGM, 6 April 1983. See also: WCRO: CR3158/3/2, AGM, 24 April 1989.

112 The Pool Fund’s separate minutes and records are in: STAFF: D5562/1/64.

113 See Leamington: WCRO: CR3068/35/1, Patient’s Handbook, 1969; Stallington: STAFF: D5562/12/1, Newsletter, September 1975; SA: MI 5524/1/20, Annual Report 1982.

114 WCRO: CR3158/3/1, Committee meeting, 5 June 1978; See: WCRO: CR3068/35/3, League Handbook, 1976; WCRO: CR3068/35/13, League Handbook, 1989.

115 WCRO: CR3158/3/1, AGM, 24 March 1982.

116 WCRO: CR3158/3/1, AGM, 18 March 1981.

117 WCRO: CR3399/1, AGM, 11 March 1957; Honorary Secretary’s AGM report, 1957; STAFF: D5562/12/1, Newsletter, September 1975.

118 WCRO: CR3894, Newsletter No. 6, June 1976.

119 See annual reports and meetings in WCA: DX-1030/1, 5 and financial correspondence in WCA: DX-1030/6.

120 SA: MI 5524/1/1–36, Annual reports 1964–96.

121 Ellis Paine *et al.*, ‘Communities as “Renewable Energy” for Healthcare Services?’

122 Beaumont, *Housewives and Citizens*, 206–8; Mold, *Making the Patient Consumer*, 25–29.

123 WCRO: CR3894, Newsletter, October 1973.

124 WAAS: 705:1336 BA12116/6, esp. press cuttings from 1980.

125 WCA: LS/LP36211, History of the Penn Hospital League of Friends.

126 WCA: DX-1030/1, AGM, 8 November 1988.

127 WCA: DX-1030/1, Extraordinary General Meeting, 16 April 1996.

128 Chamberlain’s decision to write her book on the Royal Orthopaedic Hospital’s League of Friends was inspired by this campaign. See: Chamberlain, *The Friends*.

129 WCRO: CR3174/1, AGM minutes, esp. 1981, 1985 and 1986.

130 WCRO: CR3158/3/2, AGM minutes, esp. 1989 and 1992.

131 Constitution quoted in a speech at: WCRO: CR3158/3/2, NALHF Regional Conference held in Derby, 3 April 1993.

132 Ellis Paine *et al.*, ‘Communities as “Renewable Energy” for Healthcare Services?’

133 This is made explicit in WCRO: CR3158/3/2, AGMs 1992 and 1993.

134 WCRO: CR3158/3/2, AGM, 15 July 1993.

135 WCRO: CR3158/3/2, AGM, 19 October 1994.

136 WCRO: CR3158/3/2, AGM, 15 July 1993.

137 Martin Gorsky, John Mohan and Martin Powell, ‘British Voluntary Hospitals, 1871–1938: The Geography of Provision and Utilization’, *Journal of Historical Geography*, 1999, 25, 463–82.

